# Site-Specific Material Decomposition Versus Universal Z-Effective: Optimizing Opportunistic Bone Mineral Density Screening via Dual-Energy CT

**DOI:** 10.3390/jimaging12100478

**Published:** 2026-10-01

**Authors:** Stefano Piscone, Luca Pio Stoppino, Martina Matera, Benedetta Di Millo, Giorgia Schiraldi, Paola Milillo, Roberta Vinci, Luca Macarini

**Affiliations:** Department of Medical & Surgical Sciences, Section of Diagnostic Imaging, University of Foggia, 71122 Foggia, Italy; martina.matera@unifg.it (M.M.); benedetta.dimillo@unifg.it (B.D.M.); giorgiaschiraldi1997@gmail.com (G.S.); pmilillo@ospedaliriunitifoggia.it (P.M.); roberta.vinci@unifg.it (R.V.); luca.macarini@unifg.it (L.M.)

**Keywords:** bone mineral density (BMD), dual-energy CT (DECT), dual-energy X-ray absorptiometry (DXA), osteoporosis, base material pairs, effective atomic number (Z-Effective), opportunistic screening

## Abstract

Dual-energy computed tomography (DECT) represents a high-precision tool for opportunistic bone mineral density (BMD) assessment, yet optimal algorithmic standardization across anatomical sites remains debated. This opportunistic study evaluated 60 postmenopausal female patients who underwent both dual-energy X-ray absorptiometry (DXA) and contrast-enhanced spectral DECT (80/140 kVp) within a 6-month interval. Quantitative trabecular measurements were obtained at the femoral neck and lumbar spine (L1–L4) across five distinct Base Material Pairs (BMPs) and Effective Atomic Number (Z-Effective). Material decomposition revealed significant anatomical site dependency: fat-based pairs demonstrated superior correlation with DXA BMD at the femoral neck (Calcium–Fat: *ρ* = 0.703; *p* ≤ 0.001), whereas water-based pairs performed best in the cellular, red-marrow-rich lumbar spine (CaOxMono–Water: *ρ* = 0.525; *p* ≤ 0.001). In contrast, Z-Effective functioned as a robust biomarker, maintaining significant positive linear correlations across both the proximal femur (*ρ* = 0.652; *p* ≤ 0.001) and lumbar spine (*ρ* = 0.489; *p* ≤ 0.001). Multivariable linear regression demonstrated severe multicollinearity between site-matched BMPs and Z-Effective (VIF > 5.0), confirming they capture identical physical mineral depletion. In conclusion, opportunistic DECT screening requires anatomically tailored BMP selection or, alternatively, Z-Effective alone as a unified spectral parameter to achieve reliable, accurate BMD evaluation during routine CT acquisitions.

## 1. Introduction

Osteoporosis is a systemic skeletal disorder characterized by low bone mineral density (BMD) and deterioration of the microarchitecture of bone tissue, leading to increased bone fragility and a structural predisposition to fractures following minimal trauma [1,2]. Globally, the prevalence of osteoporosis in people aged 50–85 is 21.75% [1].

Worldwide, there are 8.9 million osteoporotic fractures annually [3], representing a serious burden on public health associated with high morbidity, functional dependence and considerable healthcare costs [4,5].

The proximal femur and the lumbar spine are the main anatomical sites where catastrophic events linked to bone fragility occur [6,7]; according to the most recent estimates, the projected incidence of osteoporotic fractures of the femoral neck is set to quadruple, rising from around 1.7 million cases per year in 1990 to 6.3 million cases per year in 2050.

According to the World Health Organization (WHO), dual-energy X-ray absorptiometry (DXA) remains the established clinical gold standard for the diagnosis of osteoporosis and the prediction of fracture risk [8,9]. However, the inherently two-dimensional and projective nature of DXA imposes critical diagnostic limitations. DXA measures areal bone mineral density (BMD) (g/cm^2^) without distinguishing between cortical and trabecular bone compartments. Furthermore, areal measurements are often adversely affected by the thickness of overlying soft tissues, vascular calcifications, aortic atheromas and degenerative spinal conditions, such as osteophytosis or facet joint osteoarthritis, which frequently result in falsely elevated BMD values and the underdiagnosis of high-risk patients [10].

To overcome these physical limitations, dual-energy computed tomography (DECT) has established itself as a high-precision diagnostic alternative, making use of routine oncological CT scans without subjecting patients to additional ionizing radiation [11,12].

In particular, by using two distinct X-ray energy spectra (typically 80 kVp and 140 kVp), DECT exploits the energy-dependent differences in photoelectric absorption and Compton scattering across different tissue types [13,14]. This enables advanced tissue decomposition and a phantom-free volumetric quantification of bone mineral density (BMD) (mg/cm^3^), strictly confined within the metabolic trabecular core, whilst circumventing cortical and extra-osseous degenerative artefacts [15,16].

The diagnostic validity and algorithmic refinement of DECT-based bone densitometry have evolved rapidly in the recent literature [17].

Early studies examining manufacturer-specific hydroxyapatite–fat (HAP-fat) decomposition algorithms established that, whilst measurements of the lumbar vertebrae may be subject to variable specificity due to the complex transitions of paravertebral soft tissues, DECT of the femoral neck demonstrates robust diagnostic accuracy (82%), sensitivity (77%) and specificity (100%) compared with DXA reference standards [18]. Subsequent technical analyses have expanded this framework to systematically compare different pairs of base materials (BMPs), including calcium–fat, HAP–fat, calcium oxalate monohydrate–water (CaOxMono–water), calcium–water and HAP–water, demonstrating excellent intra-observer reproducibility and identifying the calcium–fat pair and the CaOxMono–water pair as superior correlates of areal BMD at the proximal femur [19].

Despite these methodological advances, fundamental challenges remain unresolved in current clinical practice. It is not yet clear whether a single material decomposition pair can be universally applied to disparate skeletal sites, or whether local physiological microenvironments, in particular, the prevalence of adipocyte-rich yellow marrow in the proximal femur compared with cellular red marrow with high water content in the lumbar vertebrae, require site-specific adaptation of the algorithm [20].

Furthermore, the clinical utility of CT spectral parameters, such as the effective atomic number (Z-Effective, Z-Eff), remains unvalidated, particularly with regard to its collinearity with the specific densities of the materials [21,22,23].

To address these fundamental diagnostic issues, the present study comprehensively evaluates the simultaneous performance of five distinct pairs of DECT base materials at both the femoral neck and the lumbar spine, in order to determine the exact degree of anatomical site dependence. Furthermore, we examine the diagnostic accuracy and cross-site generalizability of Z-Effective as a standardized predictor of bone mineral density (BMD) derived from DXA, with a view to defining an optimized, mathematically robust and clinically practical workflow for opportunistic screening for osteoporosis.

The aim of the study is to optimize the use of Dual-Energy CT (DECT) for opportunistic bone mineral density (BMD) screening by directly addressing the limitations of site-specific material decomposition. While the current literature explores various Base Material Pairs (BMPs), their diagnostic accuracy is inherently restricted by the specific bone and marrow microenvironment of each anatomical region—specifically, the prevalence of fat-rich yellow marrow in the proximal femur versus the cellular, water-rich red marrow in the lumbar vertebrae. By simultaneously analyzing five distinct BMPs across these two sites, this research demonstrates whether algorithmic selection must be strictly tailored to local anatomy. Consequently, the major novelty of our work is the proposition and validation of the Effective Atomic Number (Z-Effective) as a universal, cross-site biomarker. Because Z-Effective quantifies composite atomic weight without relying on predefined two-material reference pairs, it represents a unified spectral parameter capable of evaluating mineral depletion independently of anatomical location. Ultimately, by comparing site-dependent BMPs with the site-independent Z-Effective, this study aims to define a mathematically robust and clinically practical workflow for the early identification of osteopenia and osteoporosis during routine CT scans, successfully circumventing the need for additional diagnostic exams and further ionizing radiation exposure.

## 2. Materials and Methods

### 2.1. Study Population

This retrospective, opportunistic study evaluated a consecutive cohort of 60 postmenopausal female patients who had undergone both Dual-energy X-ray Absorptiometry (DXA) and contrast-enhanced Dual-Energy Computed Tomography (DECT) covering the lumbar spine and pelvis for oncological surveillance or routine clinical indications. To be eligible for inclusion, patients were required to be postmenopausal, have a maximum time interval of 6 months between DXA and DECT examinations, and present complete, artifact-free imaging coverage of both the L1–L4 lumbar vertebrae and the proximal femur.

Given the variability in the interval between examinations within the retrospectively identified cohort, an additional sensitivity analysis was performed in patients who underwent both examinations within 90 days.

Patients were excluded if they presented with a history of prior fractures, focal osteolytic or osteoblastic lesions, primary or metastatic bone tumors, or if they had underlying orthopedic hardware, prostheses, or vertebral cementoplasty.

Scans featuring severe motion or metal artifacts precluding reliable 3D volume of interest (VOI) segmentation were likewise omitted. According to the World Health Organization (WHO) diagnostic classification, DXA status was classified separately at the femoral neck and lumbar spine using the site-specific T-score: normal, T-score ≥ −1.0; osteopenia, −2.5 < T-score < −1.0; and osteoporosis, T-score ≤ −2.5. For patient-level descriptive classification, the lowest T-score across the evaluated skeletal sites was used. Site-specific classifications were used for the corresponding ROC analyses.

### 2.2. Image Acquisition Protocols

DECT examinations were acquired using a 256-slice multidetector CT system equipped with fast kVp-switching spectral imaging capability (Revolution CT, GE Healthcare, Chicago, IL, USA). Acquisitions utilized standardized Gemstone Spectral Imaging (GSI) presets featuring fast 80/140 kVp switching, a pitch of 0.984, a gantry rotation time of 0.8 s, and a fixed tube current of 200 mA managed by automatic exposure control. Monochromatic portal venous phase images (140 keV Virtual Monochromatic Images, VMI) were reconstructed at a slice thickness of 0.625 mm using standard soft-tissue convolution kernels following the intravenous administration of non-ionic contrast medium (Iohexol 350 mgI/mL at a dosage of 1.4 mL/kg) injected at a flow rate of 3.5 mL/s. Corresponding reference DXA scans were acquired using a high-resolution densitometer (Discovery A, HOLOGIC, Marlborough, MA, USA) targeting the L1–L4 lumbar spine and the proximal femur. Absolute areal BMD (g/cm^2^), along with standard T-scores and Z-scores, were calculated automatically according to established clinical criteria.

### 2.3. Post-Processing and DECT Parameter Extraction

Post-processing of spectral DECT datasets was conducted on a dedicated workstation (AW3.2, GE Healthcare). Spherical and elliptical 3D VOIs were meticulously positioned within the hyper-metabolic trabecular core of the L1–L4 vertebral bodies and the femoral neck, explicitly excluding cortical bone margins, osteophytes, subchondral sclerosis, and focal vascular structures. A standardized VOI volume of 858 mm^3^ was used at both anatomical sites. All examinations were jointly evaluated by two radiologists (L.P.S. and S.P.), with 15 and 4 years of experience in musculoskeletal imaging, respectively. Both readers assessed all cases and were blinded to the corresponding DXA results during DECT post-processing. Final measurements included in the analytical dataset were obtained by consensus between the two readers.

Quantitative density values were derived for each VOI across five distinct Base Material Pairs (BMPs), specifically Calcium–Fat, Hydroxyapatite–Fat (HAP–Fat), Carboxymonohydrated Calcium Oxalate–Water (CaOxMono–Water), Calcium–Water, and Hydroxyapatite–Water (HAP–Water). Simultaneously, the Effective Atomic Number (Z-Effective, Z-Eff) was computed for identical VOI locations.

### 2.4. Statistical Analysis

Continuous variables are expressed as mean ± standard deviation. The site-specific relationships between each DECT-derived parameter (the five BMPs and Z-Eff) and DXA-measured areal BMD (g/cm^2^) were evaluated using Spearman’s rank correlation coefficient (*ρ*). The goodness-of-fit and predictive capability of Z-Effective across anatomical sites were evaluated using simple linear regression models (R^2^). To assess the feasibility of composite predictive modeling and to detect mathematical redundancy between parameters, multiple linear regression models were constructed combining site-optimal BMPs and Z-Eff (specifically Calcium–Fat + Z-Eff for the femoral neck, and CaOxMono–Water + Z-Eff for the lumbar spine). Multicollinearity within these models was rigorously evaluated using Variance Inflation Factor (VIF) and Tolerance diagnostics. All statistical tests were two-tailed, with significance defined as *p* < 0.05. As an additional sensitivity analysis, the principal site-specific correlations were recalculated in patients with an absolute DXA–DECT interval of ≤90 days. This analysis included Calcium–Fat and Z-Effective at the femoral neck and CaOxMono–Water and Z-Effective at the lumbar spine. Spearman correlation coefficients were reported with 95% bootstrap confidence intervals and descriptively compared with the corresponding estimates from the complete cohort. Given the smaller subgroup size, emphasis was placed on the direction, magnitude, and precision of the associations rather than on statistical significance alone.

A total of six DECT-derived parameters—the five base material pairs and Z-Effective—were evaluated at each of the two anatomical sites, resulting in 12 primary correlation tests, which were considered a single family of comparisons. To control the family-wise error rate, *p*-values were adjusted using the Holm–Bonferroni procedure. Both unadjusted and Holm-adjusted *p*-values were calculated, with statistical significance defined as a two-sided adjusted *p*-value < 0.05. At each anatomical site, the parameter with the numerically highest correlation coefficient was described as the strongest observed correlate; however, numerical differences between correlation coefficients were not considered statistically significant unless formally tested.

As a secondary exploratory analysis, receiver operating characteristic (ROC) curves were constructed to assess the ability of selected DECT-derived parameters to discriminate DXA-defined osteoporosis at the corresponding anatomical site. Site-specific DXA classification was used as the reference standard, with osteoporosis defined as a T-score ≤ −2.5 at the femoral neck or lumbar spine, respectively. To limit multiplicity, ROC analysis was restricted to the site-specific parameters identified in the primary correlation analysis: Calcium–Fat and Z-Effective at the femoral neck, and CaOxMono–Water and Z-Effective at the lumbar spine. Areas under the ROC curve (AUCs) were calculated with 95% confidence intervals obtained by bootstrap resampling. Exploratory cutoff values were identified by maximizing the Youden index, and the corresponding sensitivity and specificity were calculated. Given that these thresholds were derived and evaluated within the same cohort, they were considered exploratory and were not interpreted as externally validated clinical decision limits.

## 3. Results

### 3.1. Baseline Characteristics

The final cohort consisted of 60 postmenopausal female patients. According to the World Health Organization diagnostic criteria, DXA status was classified separately at the femoral neck and lumbar spine using the site-specific T-score: normal, T-score ≥ −1.0; osteopenia, −2.5 < T-score < −1.0; and osteoporosis, T-score ≤ −2.5. Site-specific classifications were used for the corresponding ROC analyses. Thirty-three patients (55.0%) underwent both examinations within 90 days. Baseline demographics and clinical characteristics are summarized in Table 1.

### 3.2. Site-Specific Dependency of Base Material Pairs (BMPs)

Univariate Spearman correlation analysis demonstrated a clear anatomical site dependency regarding material decomposition accuracy (Table 2).

At the femoral neck, all five base material pairs showed positive correlations with local DXA BMD, all of which remained statistically significant after Holm–Bonferroni correction. Calcium–Fat showed the numerically highest correlation (*ρ* = 0.703; Holm-adjusted *p* < 0.001), closely followed by HAP–Fat (*ρ* = 0.702; Holm-adjusted *p* < 0.001) and CaOxMono–Water (*ρ* = 0.700; Holm-adjusted *p* < 0.001). Calcium–Water and HAP–Water also showed significant positive correlations. Given the nearly identical values of the three highest correlation coefficients and the absence of a formal comparison between dependent correlations, these findings should not be interpreted as demonstrating the statistical superiority of Calcium–Fat over the other material pairs.

Conversely, at the lumbar spine, all five base material pairs were positively correlated with local DXA BMD, with all associations remaining statistically significant after correction for multiple comparisons. CaOxMono–Water showed the numerically highest correlation (*ρ* = 0.525; Holm-adjusted *p* < 0.001), followed by HAP–Fat (*ρ* = 0.520; Holm-adjusted *p* < 0.001). The remaining correlation coefficients ranged from 0.480 to 0.493 and were also statistically significant after Holm–Bonferroni correction. The numerical differences among the lumbar correlation coefficients were small and were not formally tested.

### 3.3. Cross-Site Association of Z-Effective with DXA BMD

In contrast to the site-dependent performance of BMPs, Z-Effective demonstrated statistically significant positive correlations with DXA BMD at both anatomical sites, with *ρ* = 0.652 at the femoral neck and *ρ* = 0.489 at the lumbar spine. Both associations remained statistically significant after Holm–Bonferroni correction (adjusted *p* < 0.001). Although the correlation was stronger at the femoral neck, Z-Effective showed a consistent positive association with DXA BMD across both investigated sites (Table 3).

**Figure 1:**
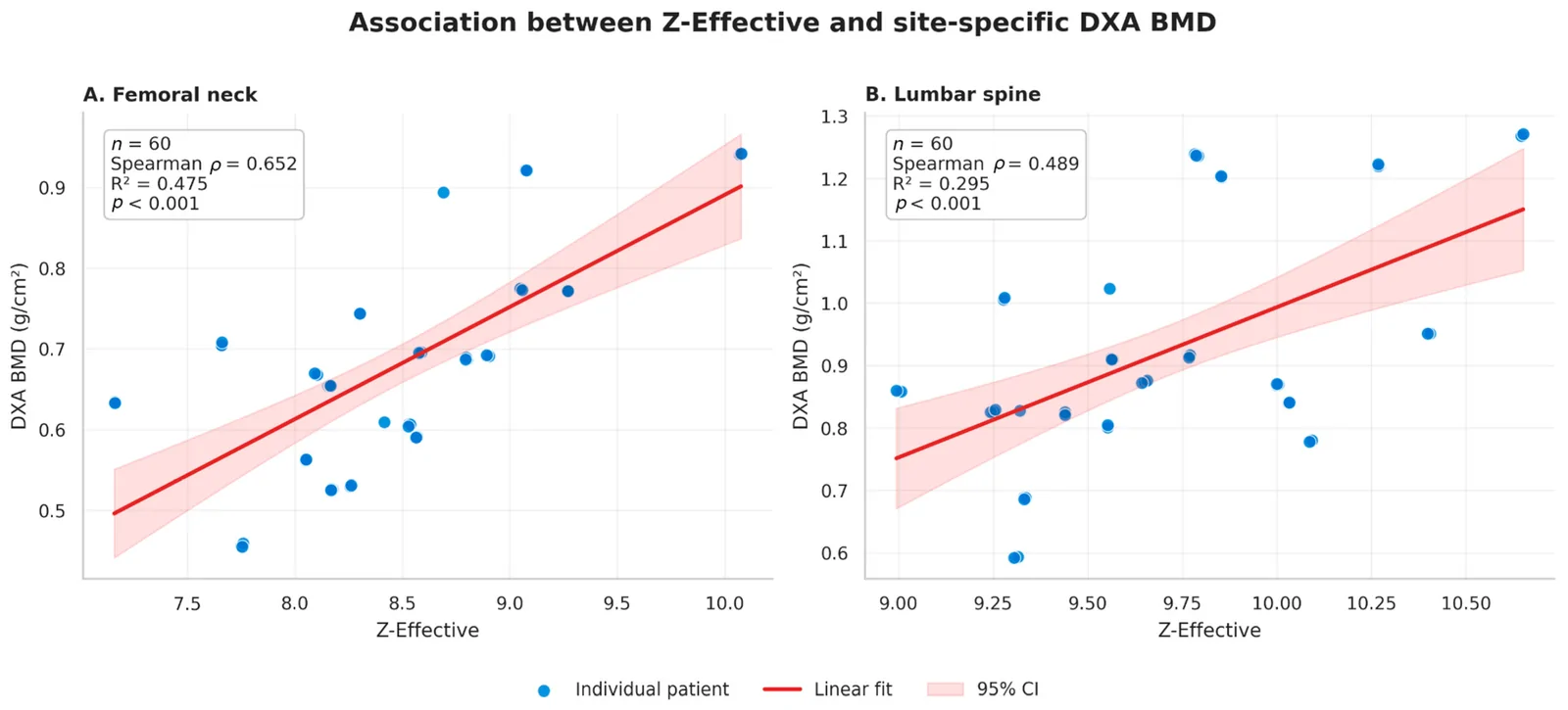
Correlation between Z-Effective and DXA BMD (g/cm^2^) at the femoral neck (**A**) and lumbar spine (**B**).

These results validate Z-Effective as a unified, site-independent parameter capable of quantifying mineral loss regardless of microenvironmental trabecular/cortical or marrow differences (Figure 1).

To explore whether combining material decomposition (BMP) and spectral metrics (Z-Eff) into a composite “DECT-Score” improved accuracy, multivariable linear regression models were constructed (Table 4).

While the overall multivariable model for the femoral neck explained 60.0% of the variance (R^2^ = 0.600, *p* ≤ 0.001) adding Z-Eff to Calcium–Fat provided no independent, statistically significant improvement.

Substantial collinearity was observed between the selected site-specific BMPs and Z-Effective. Consequently, the individual regression coefficients were estimated with limited precision, preventing the independent contribution of each predictor from being reliably distinguished within the combined models. The inclusion of both parameters did not demonstrate a clear incremental predictive contribution in this cohort. These findings indicate substantial overlap in the statistical information provided by the two parameters but do not establish that BMP measurements and Z-Effective represent the same physical quantity.

### 3.4. Exploratory Diagnostic Discrimination

Using site-specific DXA-defined osteoporosis as the reference outcome, 14 of 60 patients were classified as osteoporotic at the femoral neck and 11 of 60 at the lumbar spine. At the femoral neck, Calcium–Fat showed good diagnostic discrimination, with an AUC of 0.860 (95% CI, 0.749–0.948). An exploratory cutoff of ≤34.88 yielded a sensitivity of 92.9% and a specificity of 84.8%. Femoral Z-Effective showed an AUC of 0.771 (95% CI, 0.650–0.881), with a cutoff of ≤8.261 yielding a sensitivity of 92.9% and a specificity of 71.7%.

At the lumbar spine, diagnostic discrimination was more modest, with less precise estimates. CaOxMono–Water yielded an AUC of 0.659 (95% CI, 0.449–0.852), with an exploratory cutoff of ≤165.55 providing a sensitivity of 63.6% and a specificity of 79.6%. Lumbar Z-Effective yielded an AUC of 0.625 (95% CI, 0.426–0.809), with a cutoff of ≤9.335 providing a sensitivity of 63.6% and a specificity of 79.6%. Given the limited number of patients with site-specific osteoporosis, particularly at the lumbar spine, these thresholds should be regarded as hypothesis-generating rather than as definitive clinical cutoffs (Table 5).

### 3.5. Sensitivity Analysis Restricted to a DXA–DECT Interval of ≤90 Days

The sensitivity analysis included 33 patients who underwent DXA and DECT within 90 days. At the femoral neck, Calcium–Fat remained positively correlated with local DXA BMD (*ρ* = 0.785; 95% CI, 0.554–0.910; *p* < 0.001), as did Z-Effective (*ρ* = 0.724; 95% CI, 0.456–0.868; *p* < 0.001). Similarly, at the lumbar spine, positive correlations were maintained for both CaOxMono–Water (*ρ* = 0.565; 95% CI, 0.241–0.790; *p* < 0.001) and Z-Effective (*ρ* = 0.534; 95% CI, 0.206–0.777; *p* = 0.001). These estimates were directionally consistent with, and numerically higher than, those observed in the complete cohort, although the confidence intervals were wider owing to the smaller subgroup size.

## 4. Discussion

The rapid integration of spectral CT technology into clinical practice has revolutionized the paradigm of opportunistic bone health assessment [24]. Early diagnostic studies using single-supplier-specific material decomposition models, such as hydroxyapatite–fat (HAP-Fat), demonstrated high diagnostic performance at the femoral neck, although vertebral volumetric measurements occasionally exhibited lower specificity due to complex paravertebral structural variations and osteodegenerative overlap [18]. Subsequent technical research focused on expanding material decomposition models specifically to the proximal femur, establishing that material combinations with fat suppression or that were fat-based (e.g., Calcium–Fat) provided superior statistical alignment compared with dual-energy X-ray absorptiometry (DXA) reference standards [19].

The present study expands on this fundamental knowledge, addressing critical unresolved challenges in quantitative spectral imaging and offering significant clinical and methodological advances. Our results demonstrate that basic material pair (BMP) decomposition algorithms cannot be applied as a ‘one-size-fits-all’ post-processing solution across distinct skeletal regions. Instead, optimal quantitative accuracy requires rigorous alignment with the localized trabecular microenvironment. At the femoral neck, fat-based material pairs (calcium–fat and HAP-fat) showed superior diagnostic correlation with local areal BMD measured by DXA compared with water-based configurations. From a physiological perspective, differences in marrow composition may partially account for the site-dependent behavior of material decomposition algorithms. The adult proximal femur contains a greater proportion of fatty yellow marrow, whereas vertebral marrow retains a relatively higher proportion of cellular red marrow and water. These compositional differences may contribute to the differential performance of fat- and water-referenced decomposition approaches across anatomical sites. However, the higher correlation observed for the CaOxMono–Water pair at the lumbar spine should not be interpreted as evidence of a physiological role of calcium oxalate monohydrate in bone. In two-material DECT decomposition, basis materials primarily serve as mathematical spectral references rather than representing the actual chemical composition of the investigated tissue. Accordingly, the performance of CaOxMono–Water is more appropriately interpreted as an algorithm-dependent finding, potentially related to the water-referenced decomposition, and requires validation in larger cohorts and across different DECT platforms.

Radiologists performing opportunistic DECT screening must therefore adapt their material decomposition protocols to the anatomical region of interest.

Furthermore, whilst material decomposition algorithms require site-specific customization, the effective atomic number (Z-Eff) emerges as a robust, site-independent quantitative biomarker [21]. As Z-Effective directly calculates the composite atomic weight of a given physical volume without relying on a preselected two-material reference system, it avoids algorithm-induced selection bias. In our cohort, Z-Effective maintained strong and statistically significant correlations with DXA-derived BMD in both the proximal femur and the lumbar spine. This cross-site consistency suggests that it may represent a practical candidate parameter for opportunistic bone assessment.

The exploratory ROC analysis complements the correlation findings by providing an initial assessment of diagnostic discrimination. Calcium–Fat demonstrated good discrimination of femoral-neck osteoporosis, whereas Z-Effective showed moderate discrimination at the same site. By contrast, both CaOxMono–Water and Z-Effective showed only modest discrimination at the lumbar spine, with wide confidence intervals indicating limited precision in the estimated diagnostic performance. Differences between anatomical sites may reflect variations in marrow composition, degenerative changes affecting DXA measurements, residual effects of iodinated contrast medium, and the small number of lumbar osteoporosis cases. Accordingly, these findings provide preliminary evidence of classification performance but do not establish clinically validated diagnostic thresholds.

Given the evaluation of multiple DECT-derived parameters across two anatomical sites, the primary correlation analyses were adjusted using the Holm–Bonferroni procedure. All associations remained statistically significant after correction, reducing the likelihood that the observed relationships were attributable solely to multiple testing. Nevertheless, statistical significance should not be interpreted as evidence of superiority among material pairs. Several correlation coefficients were very similar, particularly at the femoral neck, and no formal comparisons between dependent correlations were performed. Accordingly, references to the “best-performing” parameter in this study denote the numerically strongest observed association rather than statistically demonstrated superiority.

The multivariable analysis showed substantial collinearity between the selected site-specific BMPs and Z-Effective. This finding indicates that, within the present cohort and under the investigated acquisition conditions, the two parameters provided overlapping information related to DXA BMD. Consequently, their independent regression coefficients could not be estimated with high precision, and combining the parameters did not demonstrate a clear incremental predictive contribution. However, statistical collinearity does not imply physical identity or complete clinical interchangeability.

Base-material decomposition values depend on the selected reference materials and the corresponding vendor-specific decomposition model, whereas Z-Effective is a composite estimate derived from the energy-dependent attenuation characteristics of the sampled tissue. The two approaches may therefore respond differently to marrow composition, pathological marrow infiltration, contrast enhancement, and technical acquisition factors. Our results support statistical overlap within this specific cohort but do not establish that BMP measurements and Z-Effective quantify the same underlying process.

Temporal discordance between DXA and DECT represents a potential source of measurement variability, particularly if bone-active treatment was initiated or modified between examinations. Nevertheless, in the sensitivity analysis restricted to the 33 patients who underwent both examinations within 90 days, all four principal site-specific associations remained positive and statistically significant.

The clinical implications of opportunistic DECT screening are substantial, particularly for cohorts of cancer patients or those with chronic diseases undergoing longitudinal CT surveillance [23]. The use of existing spectral CT scans enables the early diagnosis of subclinical osteopenia and osteoporosis, facilitating timely pharmacological intervention to prevent catastrophic fragility fractures without exposing patients to additional ionizing radiation or the financial burden associated with separate DXA scans.

However, certain limitations must be acknowledged. The sample size (*N* = 60), whilst statistically robust for preliminary validation, requires further expansion in larger, multicenter prospective cohorts to establish reference thresholds stratified by age and sex. Furthermore, all DECT examinations were acquired during the portal venous phase following intravenous administration of iodinated contrast medium. Iodine accumulation within the vertebral bone marrow may influence spectral material-decomposition and Z-Effective measurements, particularly at the lumbar spine. In the absence of independent quantification of iodine concentration and paired unenhanced acquisitions, the magnitude and direction of this potential effect could not be determined. Consequently, the lumbar findings should be interpreted with caution and cannot be assumed to be directly transferable to unenhanced DECT examinations.

Future prospective studies should compare paired contrast-enhanced and unenhanced acquisitions and quantify marrow iodine concentration to determine its influence on BMP and Z-Effective measurements [24].

The ROC-derived thresholds were selected and evaluated within the same relatively small cohort, without internal cross-validation or validation in an independent external dataset. Their apparent sensitivity and specificity may therefore be optimistic. These cutoffs should be regarded as exploratory and require prospective external validation before clinical implementation.

Demographic and anthropometric variables, including age, body weight, height, and BMI, were not available in the analytical dataset. These factors may influence both DXA BMD and bone marrow composition and could therefore confound the observed associations. Consequently, the reported relationships are unadjusted, and residual confounding cannot be excluded. Future prospective studies should systematically collect these variables and assess whether the associations between spectral DECT parameters and DXA BMD remain independent after adjustment for age and body composition.

Some material decomposition algorithms and spectral post-processing software tools remain vendor-specific. Variations in tube voltage switching modes, dual-source configurations and detector-based spectral CT architectures across different manufacturers could influence absolute density values and Z-Eff results, making standardization across suppliers a fundamental requirement for large-scale clinical adoption [25].

Finally, formal intra- and interobserver reproducibility was not assessed. Larger prospective multicenter studies including more diverse populations, independent readers, and external validation cohorts are required.

## 5. Conclusions

Opportunistic assessment of BMD using dual-energy CT shows high diagnostic performance, but post-processing algorithms must take local bone microenvironments into account. When using base material pairs, optimal material decomposition requires targeting the site-specific physiological composition: fat-based pairs (Calcium–Fat/HAP-Fat) for the proximal femur, which is rich in yellow marrow, and water-based pairs (CaOxMono-Water) for the lumbar spine, which is rich in red marrow.

Alternatively, the effective atomic number (Z-Effective) could serve as a biomarker independent of anatomical location, capable of delivering diagnostic performance for both the spine and the hip. The substantial collinearity between BMP measurements and Z-Effective indicates overlapping predictive information in this cohort: both parameters could be used individually to carry out opportunistic screening during routine CT scans, without subjecting patients to additional radiation. Our findings support the potential use of selected DECT-derived parameters for opportunistic BMD assessment, particularly at the femoral neck.

## Figures and Tables

**Table 1:** Site-Specific DXA Classification of the Study Cohort (*N* = 60).

Characteristic	Normal (T-Score ≥ −1.0)	Osteopenia (−2.5 < T-Score < −1.0)	Osteoporosis (T-Score ≤ −2.5)	Total Cohort
Number of patients (F)	19	27	14	60
Number of patients (LV)	21	28	11	60

**Table 2:** Site-Specific Spearman Correlation Between DECT Base Material Pairs and DXA BMD.

Base Material Pair (BMP)	Femoral Neck (vs. DXA Femoral BMD)		Lumbar Spine (vs. DXA Lumbar BMD)	
	Spearman’s Rho (*ρ*)	*p*-Value	Spearman’s Rho (*ρ*)	*p*-Value
Calcium–Fat	0.703	<0.001	0.493	<0.001
HAP–Fat	0.702	<0.001	0.520	<0.001
CaOxMono–Water	0.700	<0.001	0.525	<0.001
Calcium–Water	0.672	<0.001	0.490	<0.001
HAP–Water	0.661	<0.001	0.480	<0.001

**Table 3:** Site-Specific Spearman Correlation Between Z-effective and DXA BMD.

Parameter	Femoral Neck (vs. DXA Femoral BMD)		Lumbar Spine (vs. DXA Lumbar BMD)	
	Spearman’s Rho (*ρ*)	*p*-Value	Spearman’s Rho (*ρ*)	*p*-Value
Z-Effective	0.652	<0.001	0.489	<0.001

**Table 4:** Multivariable Linear Regression and Collinearity Diagnostics.

Anatomical Model	Predictors	Adjusted R^2^	Standardized Coefficient (*β*)	*p*-Value	Tolerance	VIF
Femoral Neck	Overall Model	0.600	-	<0.001	-	-
	Calcium–Fat (F)		0.540	0.021	0.15	>5.0
	Z-Effective (F)		0.280	0.180	0.15	>5.0
Lumbar Spine	Overall Model	0.330	-	0.045	-	-
	CaOxMono-Water (LV)		0.410	0.120	0.20	>5.0
	Z-Effective (LV)		0.290	0.250	0.20	>5.0

**Table 5:** AUC, area under the receiver operating characteristic curve; CI, confidence interval; DXA, dual-energy X-ray absorptiometry; DECT, dual-energy computed tomography. Osteoporosis was defined using the site-specific DXA T-score threshold of ≤−2.5. Confidence intervals were estimated using 5000 bootstrap resamples. Cutoffs were selected by maximizing the Youden index and should be regarded as exploratory because they were derived and evaluated in the same cohort.

Anatomical Site	DECT Parameter	AUC (95% CI)	Exploratory Cutoff	Sensitivity	Specificity
Femoral Neck	Calcium–Fat (F)	0.860 (0.749–0.948)	≤34.88	92.9%	84.8%
	Z-Effective (F)	0.771 (0.650–0.881)	≤8.261	92.9%	71.7%
Lumbar Spine	CaOxMono-Water (LV)	0.659 (0.449–0.852)	≤165.55	63.6%	79.6%
	Z-Effective (LV)	0.625 (0.426–0.809)	≤9.335	63.6%	79.6%

## Data Availability

The data presented in this study are available on request from the corresponding author due to privacy or ethical restrictions.
